# After the Prescription: The Clinical Support Gap in Telehealth-Based GLP-1 Care

**DOI:** 10.2196/101874

**Published:** 2026-05-28

**Authors:** Anna Zucker

**Keywords:** digital health, telehealth, GLP-1, semaglutide, tirzepatide, obesity, weight loss, glucagon-like peptide-1 agonist

## Abstract

GLP-1 medications offer promise for obesity management and are increasingly accessible via digital platforms. In this *News and Perspectives* article, JMIR Correspondent Anna Zucker reports on the gap in clinical support that could undermine their potential benefits.

**Key Takeaways:**

- Telehealth platforms have significantly increased access to GLP-1 medications for people with obesity, making prescriptions faster and easier to obtain and helping millions lose weight and improve their health, though financial barriers remain for some.
- These platforms don’t always offer nutrition, exercise, and behavioral change guidance required to help patients lose weight safely. As a result, research finds that some patients lose lean tissue and experience micronutrient deficiencies.
- Integrating registered dietitians and other health professionals into telehealth programs can help patients make the sustainable changes necessary to maximize the benefits of GLP-1 medications.

Obesity affects 40% of Americans and increases the risk of life-threatening conditions, including hypertension, type 2 diabetes, and heart disease. The general advice to “eat less and exercise more” oversimplifies a complex metabolic condition. Glucagon-like peptide-1 agonist (GLP-1) medications are now helping transform it.

These medications are powerful tools, but not complete cures. As telehealth platforms make GLP-1 medications more widely available, a gap emerges between the benefits of prescription access and the drawbacks of insufficient clinical support. Emerging research has found that patients are losing muscle along with fat, a risk that becomes more dangerous with age.

## Telehealth Changes Who Gets GLP-1 Treatment

GLP-1 medications, including semaglutide (Ozempic and Wegovy) and tirzepatide (Mounjaro and Zepbound), have revolutionized treatment for both obesity and type 2 diabetes. They mimic hormones naturally produced by the body to suppress appetite, increase feelings of fullness, and reduce food cravings. Research suggests they have comparable effects to bariatric surgery, with an average loss of 20% of body weight in clinical trials. Beyond weight loss, research suggests they may also help with cardiovascular, kidney, and liver conditions.

Once only accessible to type 2 diabetes patients under specialist care, telehealth platforms now make them available to anyone with a BMI classifying them as overweight or obese. Real-world results back up the effectiveness of telehealth. A large study of over 50,000 participants using telehealth-delivered GLP-1 medications yielded results similar to those of major clinical trials. Participants lost 8.9% of their weight within 3 months, and 19% at 12 months. A smaller 2025 study found that participants who followed a 50-day telehealth program with liraglutide lost an average of 4.9 kilograms, with high adherence and interest in continuing treatment.

For self-pay patients, cost remains a barrier. In one study (currently available as a preprint), telehealth patients on semaglutide achieved weight loss comparable to clinical trial benchmarks, but those on tirzepatide didn’t meet the same standard, potentially due to the higher cost of a clinical dosage of tirzepatide.

## When Prescribing Moves Faster Than Clinical Support

Despite the benefits of telehealth for GLP-1s, research suggests that one of the biggest potential drawbacks is the lack of ongoing clinical support for patients.

A 2026 scoping review in *Obesity Reviews* examined 12 clinical trials on patients taking GLP-1 medications and their nutrient intake. While participants reduced caloric intake by 24% to 39% and saw significant weight loss, 40% of that weight was lean tissue. Only three of the 12 studies involved a registered dietitian (RD), and one study found nutrient inadequacies. The authors caution that very low caloric intake without clinical guidance may “increase the risk of inadequate protein intake, dietary quality, and micronutrient deficiencies.” The loss of lean mass exceeds the natural rate of muscle loss in older adults.

Sarah Skobeloff, MS, RD, LDN, a registered dietitian who works with patients taking GLP-1 medications, finds her clinical experience reflects this. “Appetite suppression can cause a person to consume too few calories relative to their maintenance needs. Rapid weight loss is more likely to cause muscle loss, putting my clients at risk for osteopenia and osteoporosis.” She adds, “Decreased energy is often a side effect I see because if your body doesn’t get enough calories, specifically carbohydrates, you will have less energy. Clients risk nutrient deficiencies like vitamin D and calcium, which are essential for maintaining bones.”

Two telehealth patients reported similar experiences. Cheri says, “I did a lot of personal research. I joined Facebook support groups and learned a lot. I will say the telehealth companies need to educate you more on nutrition.” She had hair loss during her treatment, which she assumes was due to rapid weight loss and insufficient protein. An anonymous patient—also a nurse practitioner (NP)—experienced fatigue, which she attributed to insufficient calories.

## More Access, Insufficient Care

Increased access to GLP-1 medications can lead to major health improvements for more people, but without nutritional support, it can also increase the risk of adverse outcomes. Research highlights that the primary dietary advice during GLP-1 therapy is calorie reduction, with insufficient focus on adequate nutrition.

Skobeloff describes the tension clearly: “The major difference between telehealth and a traditional clinical setting is that the client receives interdisciplinary team care. You can’t have interdisciplinary team care (PCP, cardiologist, endocrinologist) when using an online company.”

**Figure:**
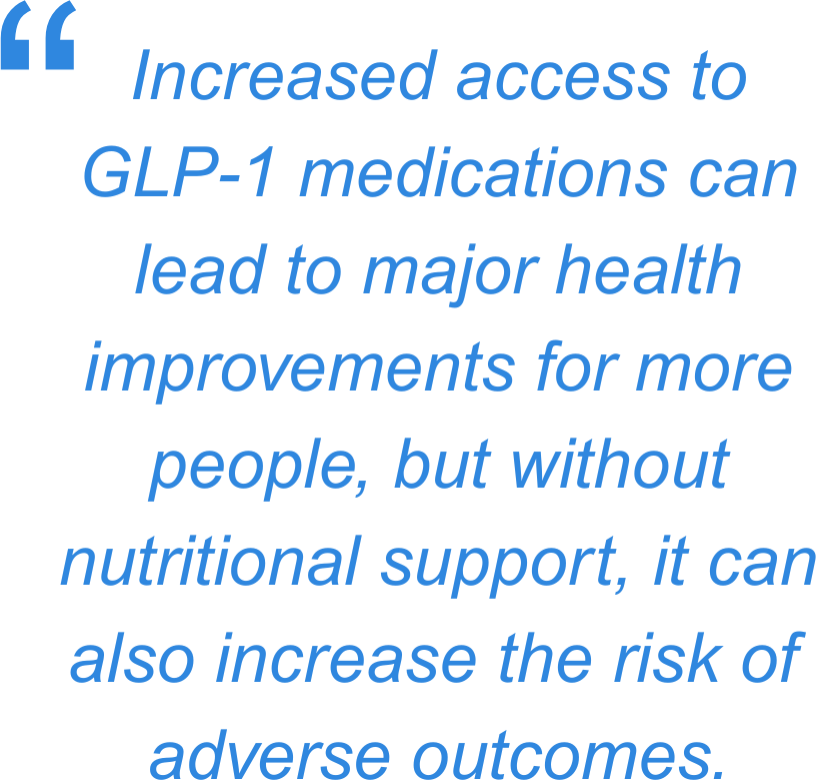


The anonymous NP, who also works at a clinic managing weight-loss patients, offers in-person and telehealth visits and illustrates the contrast. “I see patients for a 30-minute visit once a month. I cover every aspect of their weight management care.” Their husband, on the other hand, has received GLP-1 therapy through a telehealth platform for 3 years. “He has had no communication with a provider outside of a 3-minute call when the medication was initially ordered. No contact or follow-up since then.”

## Looking Ahead

The authors of the *Obesity Reviews* study recommend early dietitian involvement, a nutrient-dense, high-protein diet (1.2‐1.5 grams per kilogram of body weight per day), ongoing monitoring for nutrition deficiencies, and psychological support for emotional or disordered eating. They suggest a hybrid model to pair telehealth prescriptions with clinical support and reimbursement for seeing a dietitian.

Some platforms are already on board. One telehealth patient, Lisa, uses an app that includes monthly visits with an RD. “She answers all my questions and gives me incredible guidance on what to eat and when.” Lisa shares, “The medication itself is a great motivator and tool, but it’s not a miracle cure.”

Skobeloff agrees and adds that nutrition support is only part of the equation for muscle retention. “Providers need to rely on their allied health professionals to deliver the best patient care. They need to engage with dietitians to prevent nutrient deficiencies, with exercise physiologists to ensure weight-lifting is programmed correctly, and with therapists to ensure their relationships with their body and food are strong. A supportive community results in better patient outcomes. Prescribers need a patient-centered approach, considering all aspects of health, not just weight.”

More access to GLP-1 medication through telehealth is helping people achieve meaningful changes that may have felt impossible before. To make that change sustainable, we need integrated clinical care to support that access.

